# Derivative-based time-adjusted analysis of diurnal and within-tree variation in the OJIP fluorescence transient of silver birch

**DOI:** 10.1007/s11120-023-01033-x

**Published:** 2023-06-29

**Authors:** Olusegun Olaitan Akinyemi, Jaroslav Čepl, Sarita Keski-Saari, Ivana Tomášková, Jan Stejskal, Sari Kontunen-Soppela, Markku Keinänen

**Affiliations:** 1 Department of Environmental and Biological Sciences, University of Eastern Finland, Yliopistokatu 7, P.O. Box 111, 80101 Joensuu, Finland; 2 Department of Genetics and Physiology of Forest Trees, Czech University of Life Sciences Prague, Kamýcká 129, 165 00 Prague 6, Czechia; 3 Center for Photonics Sciences, Yliopistokatu 7, P.O. Box 111, 80101 Joensuu, Finland

**Keywords:** Chlorophyll fluorescence, JIP test, Photosynthesis, Diurnal variation, Within-tree variation, *Betula pendula*

## Abstract

The JIP test, based on fast chlorophyll fluorescence (ChlF) kinetics and derived parameters, is a dependable tool for studying photosynthetic efficiency under varying environmental conditions. We extracted additional information from the whole OJIP and the normalized variable fluorescence (*V*_*t*_) transient curve using first and second-order derivatives to visualize and localize points of landmark events. To account for light-induced variations in the fluorescence transient, we present a time-adjusted JIP test approach in which the derivatives of the transient curve are used to determine the exact timing of the J and I steps instead of fixed time points. We compared the traditional JIP test method with the time-adjusted method in analyzing fast ChlF measurements of silver birch (*Betula pendula*) in field conditions studying diurnal and within-crown variation. The time-adjusted JIP test method showed potential for studying ChlF dynamics, as it takes into account potential time shifts in the occurrence of J and I steps. The exact occurrence times of J and I steps and other landmark events coincided with the times of significant differences in fluorescence intensity. Chlorophyll fluorescence parameters were linearly related to photosynthetic photon flux density (PPFD) at different times of day, and the values obtained by the time-adjusted JIP test showed a stronger linear regression than the traditional JIP test. For fluorescence parameters having significant differences among different times of day and crown layers, the time-adjusted JIP test resulted in more clear differences than the traditional JIP test. Diurnal ChlF intensity data indicated that differences between the southern and northern provenance were only evident under low light conditions. Taken together, our results emphasize the potential relevance of considering the time domain in the analysis of the fast ChlF induction.

**Supplementary Information:**

The online version contains supplementary material available at 10.1007/s11120-023-01033-x.

## Introduction

The JIP test (Strasser et al. 2000, 2004) based on fast chlorophyll fluorescence (ChlF) induction dynamics (OJIP transient) has become an important tool for analyzing photosynthetic processes (Maxwell and Johnson 2000; Force et al. 2003; Stirbet et al. 2018) and plant’s responses to varying environmental conditions (Baker and Rosenqvist 2004). Calculated ChlF parameters and the OJIP transient curvature have been reported to show a high sensitivity to non-optimal environmental conditions, including variation in light quality (Tsimilli-Michael and Strasser 2001; Stirbet and Govindjee 2012), nutrient and water deficiency (Zivcak et al. 2014; Sperdouli and Moustakas 2012), extreme temperatures (Zushi et al. 2012), and ozone exposure (Bussotti et al. 2011). The ChlF transient can also be considered a “fingerprint” of plant’s physiological responses to environmental signals (Tyystjärvi et al. 1999).

The OJIP transient has four important steps: O–J–I–P from the start of the induction to the maximal fluorescence. Conventionally, the fluorescence analysis and the JIP test are characterized by fixed times of occurrence for the J (2 ms) and I (30 ms) steps (Strasser et al. 1995; Zushi et al. 2012; Chen et al. 2014; Zushi and Matsuzoe 2017; Guo et al. 2020; Koutra et al. 2022)**.** According to Kalaji et al. (2017), using 2 ms for the J-step provides variability that indicates stress, whereas kinetically, 3 ms is more accurate for the electron transport chain. Essentially, variations along the fluorescent transient indicate changes in energy and electron transport processes within the photosystems (Kalaji et al. 2011; Zivcak et al. 2015; Khan et al. 2020). In the analysis of the fast ChlF induction, emphasis has been given to differences in fluorescence intensities at the steps or the amplitudes between different steps, although rate constants and half-times of the phases are also used. Notably, differences in the time domain and the variation in the occurrence time of intermediate steps have gained less attention.

For studies utilizing the OJIP transient, shifts in the position of the steps should be expected, especially under heterogeneous conditions in the field (Khan et al. 2020). Every day, leaves are exposed to varying light intensity and quality due to changes in sun angle, weather conditions, and wind-induced canopy movement (Way and Pearcy 2012). Prevailing light conditions can trigger different structural, functional, and pigment-level adaptive response mechanisms (Lichtenthaler et al. 1981; Urban et al. 2007), resulting in optimization of light perception and foliage photosynthetic capacity. Particularly, within a tree crown, sun and shade leaves follow different strategies. Shade leaves are typically thin, large, and have high mass-based chlorophyll content to maximize light absorption with the minimum cost of maintaining excess photosynthetic machinery (Boardman 1977; Evans 1989; Pons and Pearcy 1994; Mathur et al. 2018). By contrast, sun leaves are typically smaller and thicker, with well-developed palisade parenchyma and high photosynthetic efficiency to utilize the abundant light energy while avoiding photoinhibition caused by excess irradiance (Rijkers et al. 2000; Lichtenthaler et al. 2007; Mathur et al. 2018). For optimum plant photosynthetic efficiency in natural environments, the capacity to detect and respond to spatial and temporal variation in light conditions is vital. Thus, the physiological conditions of tree leaves may differ depending on environmental factors such as temperature and light intensity. This is manifested in the kinetics of the OJIP transient and may result in an earlier or later occurrence time of the J and I steps (Stirbet and Govindjee 2012).

Aside from the well-established and widely used JIP test parameters (Strasser et al. 2000, 2004), the information granted from the whole transient curve has been used to assess species-specific or light intensity-modified times of occurrence for the J and I steps (Tomek et al. 2001; Xia et al. 2019; Khan et al. 2020). To determine the appearance of the J and I steps, Tomek et al. (2001) used the second derivative, and Xia et al. (2019) used a derivative-based curvature function. In addition, other intermediate steps have been determined, such as the L and K steps at the O–J rise in stressed plants (Strasser et al. 2004; Bednaříková et al. 2020), and the G and H steps at the I–P phase (Tsimilli-Michael et al. 1999, Strasser et al. 2004, Bednaříková et al. 2020). The OJIP curve has also been analyzed using various normalizations (Strasser et al. 2004; Stirbet and Govindjee 2011; Kalaji et al. 2014a, b) and subtractions or double-normalized difference kinetics (Strasser et al. 2007; Tsimilli-Michael 2019).

Here, we present a derivative-based approach to analyze the OJIP fluorescence transient. First and second-order derivatives of the fluorescence curve are used to determine the occurrence time of the J and I steps and other landmark events defined by alterations in the rate of change of fluorescence intensity. The fluorescence intensities at the derivative-defined appearance times of the J and I steps and at the conventional fixed times of occurrence are then used to compare the time-adjusted and traditional JIP tests. The performance of the two approaches for the JIP test is evaluated by studying the light dependency of ChlF kinetics for Finnish silver birch (*Betula pendula*) genotypes originating from the northern and southern provenances (latitude of origin 67°N and 62°N, respectively) grown in a common garden at a latitude of 62°N. We tested the ability of the two methods to assess diurnal and within-crown ChlF variation. In addition, we studied the variation in the occurrence time of the J and I steps and other landmark events defined using derivatives and compared the results to the variation in the fluorescence intensities. We had the following hypothesis:

1. Locations determined by the derivatives of the fluorescence transient, including steps J and I, coincide with the times of significant differences in fluorescence intensity.
2. Using fluorescence intensity values for J and I steps at their determined times of occurrence decreases the risk of false negative results in statistical testing of the JIP test parameters.
3. Using fluorescence intensity values for the J and I steps at their determined times of occurrence strengthens the relationship of light with computed fluorescence parameters.
4. Utilizing the whole range of the transient can be used as a fingerprint for classification, such as between provenances or times of day.

## Materials and methods

We used the data from two separate experiments to explore the OJIP transient full range and to compare the time-adjusted JIP test and the widely used traditional JIP test for analyzing fast-fluorescence kinetics data. The first experiment examined diurnal variation in ChlF kinetics between two silver birch provenances, and the second examined the within-crown variation of ChlF kinetics in silver birch. Both experiments were executed in the field at varying light levels.

### Study site and plant materials

Five-year-old silver birch (*Betula pendula* Roth*)* trees of two provenances, a northern (Kittilä, 67°N) and a southern (Vehmersalmi, 62°N) one, growing in a common garden in Joensuu, Finland (62°N) were used in this study. Vehmersalmi is situated in central Finland, but silver birch trees from Vehmersalmi belong to the southern group of genotypes in previous studies (Deepak et al. 2020; Tenkanen et al. 2020, 2021) and therefore are referred to as southern provenance in this study. Micropropagated cloned replicates from naturally regenerated stands were planted in July 2010. For details on the common garden experimental design and set-up, see Heimonen et al. (2015). For the environmental parameters in the field and related to the places of origin, see Tenkanen et al. (2020).

### Leaf measurements

In the first experiment, diurnal ChlF variation was measured on July 16, 2015. Measurements were taken on three randomly selected cloned trees for two genotypes: K1, originating from Kittilä (67°N) and V14, originating from Vehmersalmi (62°N). The height of the trees was approximately 4–5.5 m for the northern and southern provenances, respectively, during this study. The adaxial side of fully mature short-shoot leaves was used. The short-shoot leaves burst during spring at the same time (Maillette 1982; Deepak et al. 2019). Leaves were sampled in the middle of branches at breast height, one leaf per tree. The same leaf was measured from all six individuals at dawn (6 h), morning (10 h), midday (14 h), evening (18 h), and night: (22 h). Thirty measurements were taken (5 times × 2 genotypes × 3 trees). Photosynthetic photon flux density (PPFD, μmol m^−2^ s^−1^) measurements were made using the ceptometer integrated into Fluorpen FP 100 (Supplementary Table S1).

In a second experiment, within-crown ChlF variation was measured on 7th and 28th July 2015, between 10:00 and 12:00, on three trees of approximately the same height (5.5 m) from genotype Vehmersalmi (62° southern provenance). Measurements were taken at the bottom (B, 0.9 m), middle (M, 2.3 m), and top (T, 3.7 m) parts of the crown and four cardinal sides of the trees (north, east, south, west). A total of twelve leaves were measured per tree. Leaves were sampled from the middle of the branch. A different leaf in the same branch was used on both sampling days. In total, 72 measurements were taken, i.e., (2 days × 3 vertical crown positions × 4 cardinal positions × 3 trees).

### Chlorophyll fluorescence kinetics measurements

In both experiments, we measured ChlF kinetics using FluorPen FP100 (PSI, Czech Republic) after the selected leaf was dark-adapted for 30 min with leaf clips shielding it from ambient light (Kalaji et al. 2014a, b). Leaves were accessed using a twin-step platform ladder (height 5 m) at the study site. The leaves were not removed from the trees for the measurements. The default set-up of the OJIP—program in FluorPen FP100 was used to measure the minimal fluorescence intensity (*F*_0_)_,_ maximal fluorescence intensity (*F*_*m*_), and calculated parameters (Strasser et al. 2000, 2004, Bussotti et al. 2010, Stirbet and Govindjee 2011) of the OJIP test (Definitions and derivations of these parameters are available as Supplementary Table S2).

### Statistical analysis

All data processing and statistical analyses were conducted using R software (R Core Team 2021).

### First and second-order derivatives of the OJIP and Vt transient

Derivatives were determined on the double-normalized relative variable fluorescence data (*V*_*t*_ transients), calculated as *V*_*t*_ = (*F*_*t*_ − *F*_0_)/(*F*_*M*_ − *F*_0_). Cubic smoothing spline was fitted into data using *smooth.spline* function from base *R* with default parameters. To resample values from fitted spline we used *predict.smooth.spline* function with a logarithmic sequence of time points, regularly spaced along a logarithmic time scale generated by the following equation:

1 $$\left\{{t}_{n}\right\}{=\left\{{10}^{(1+n*0.005)}\right\}}_{n=1}^{1061}$$

Approximation of first and second-order derivatives was then calculated as differences between adjacent points from this logarithmic time sequence using base *R* diff function with differences parameter equal to 1 for first-order derivative and 2 for second-order derivative.

Local maxima and minima of first-order derivatives allowed us to estimate the position of the inflection points in the rise of the fluorescence at the O–J, J–I, and I–P phases (local maxima) or the plateaus after the J- and I-step (local minima) for each curve. Finally, the local minima of the second-order derivative allowed us to identify positions of inflection points defined as J and I (Tomek et al. 2001). A detailed description of the approach is available as Supplementary file 2 or at http://3.70.185.46:3839/ojip_vignette/.

### Nonparametric repeated-measures model

A nonparametric model for repeated-measures analyses was performed with the *nparLD *function within the nparLD package (Noguchi et al. 2012), where F1 LD F1 model was fitted for the diurnal experiment and F2 LD F1 model for the crown experiment. In all cases, the dependent value was either a parameter from the JIP test or the value of fluorescence and *V*_*t*_ at a given time point. We applied the model to each time point along the transients. In the diurnal experiment, we tested the effects of time of day as repeated-measures, provenance, and interaction of time of day and provenance. In the within-crown variation experiment, we tested the impact of sampling dates as repeated-measures and factor of crown layers, crown sides, and interaction of crown layers and crown sides. To see statistical significance of difference among crown positions for chosen indices in separated sampling days, we fitted the following linear mixed model with asreml package (Butler et al. 2017):

$${\varvec{Y}} = {\varvec{1}}\mu + {\varvec{Xa}} +{\varvec{Zb}} + {\varvec{e}}.$$

where ***Y ***corresponds to the data vector; *μ* is the overall mean effect; ***a ***is the fixed vector of crown strata; ***b ***is the random vector of individual clones, and ***e ***is the random vector of errors, with letters ***X ****and**** Z ***designate incidence matrices for fixed resp. random effects, and ***1 ***is a vector of ones. Wald test (performed by *wald* function from asreml package) was used to obtain significances. The non-parametric repeated-measures model was also applied in testing for statistical differences in times of occurrence of the intermediate steps of the transients among times of day and between provenances and crown layers.

### Comparison of the time-adjusted JIP test with the traditional JIP test

In both experiments, JIP test fluorescence parameters were computed with fluorescence intensity values at J and I steps either at fixed timepoints at 2 ms and 30 ms, respectively, and at times determined by the derivative approach.

### PCA, PLS-DA and regression of fluorescence parameters with PPFD

Principal component analysis (PCA) was performed on all data points of the *V*_*t*_ transient in the diurnal experiment using the *prcomp* function implemented in base *R*. Partial least-squares discriminant analysis (PLS-DA) was performed on the same data using the *plsda* function with a grouping of time of day but not provenance. Variable importance regarding these groupings was extracted using the *vip* function; both implemented in the mixOmics package (Rohart et al. 2017).

Linear regression (lm function from base R) was used to display the relationship between fluorescence parameters and PPFD at different times of day and with PLS-DA group centroids. Slope, intercept, *R*^2^, and standard error of the regression slope using the time-adjusted JIP test and traditional JIP test approaches are reported for provenances separately.

## Results

**Fig. 1:**
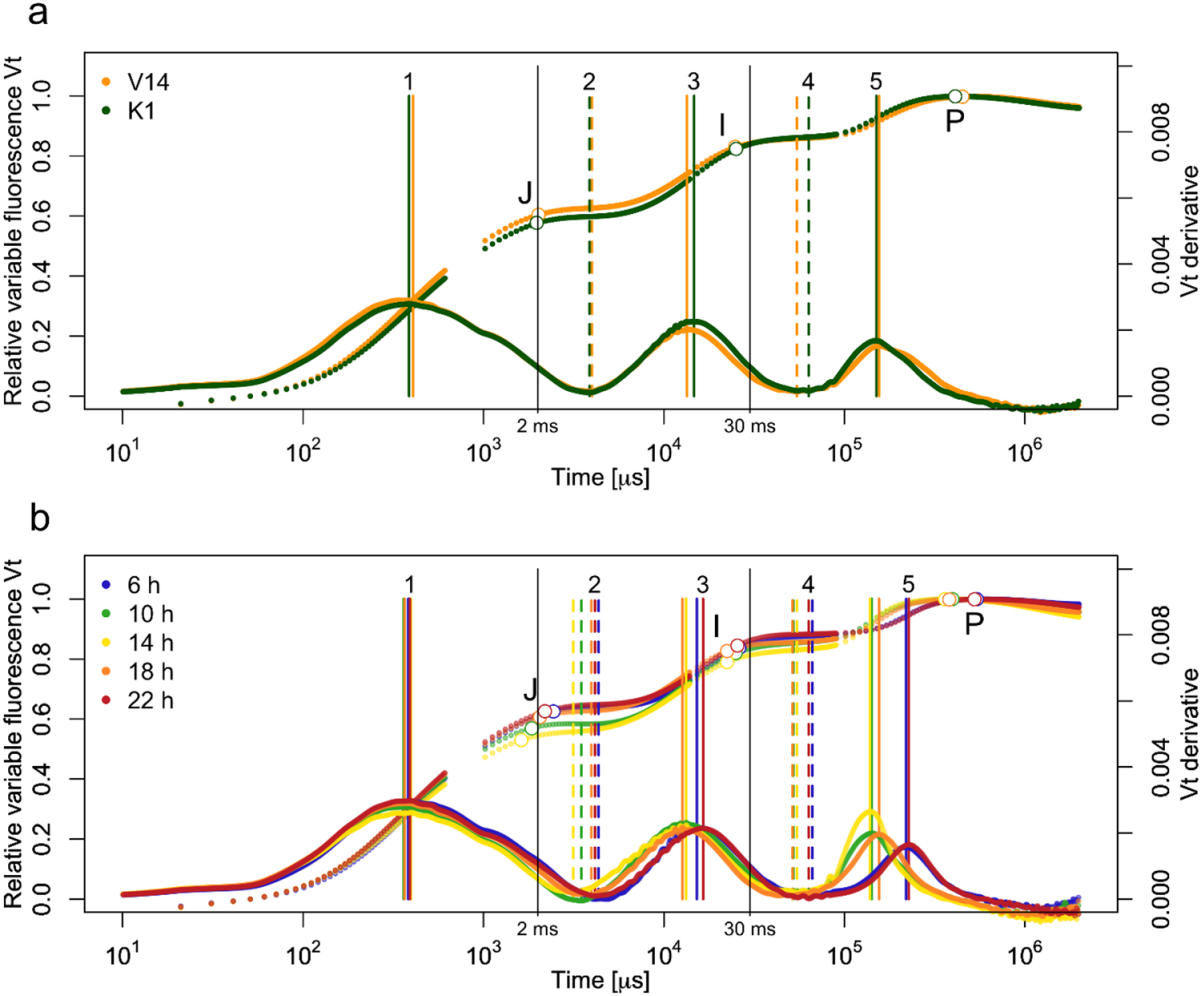
Curves of relative variable fluorescence (*V*_*t*_) transient of silver birch provenances from northern (67°N, K1) and southern (62°N, V14) Finland measured at five times of day. Upper curves show *V*_*t*_ transient for different provenances (**a**) and times of day (**b**). Lower curves show the 1st derivative of *V*_*t*_ transient. Mean curve of each provenance (**a**) or time of day (**b**) are displayed, with vertical lines showing the positions of plateaus and inflection points (locations 1–5) for each provenance or time of day separately. Positions of the J and I steps, for the time-adjusted JIP analysis, are marked as small circles in the curves, while positions of the J (2 ms) and I (30 ms) steps for the traditional JIP method are shown. The combined OJIP curve of the two provenances is shown for the diurnal variation

**Fig. 2:**
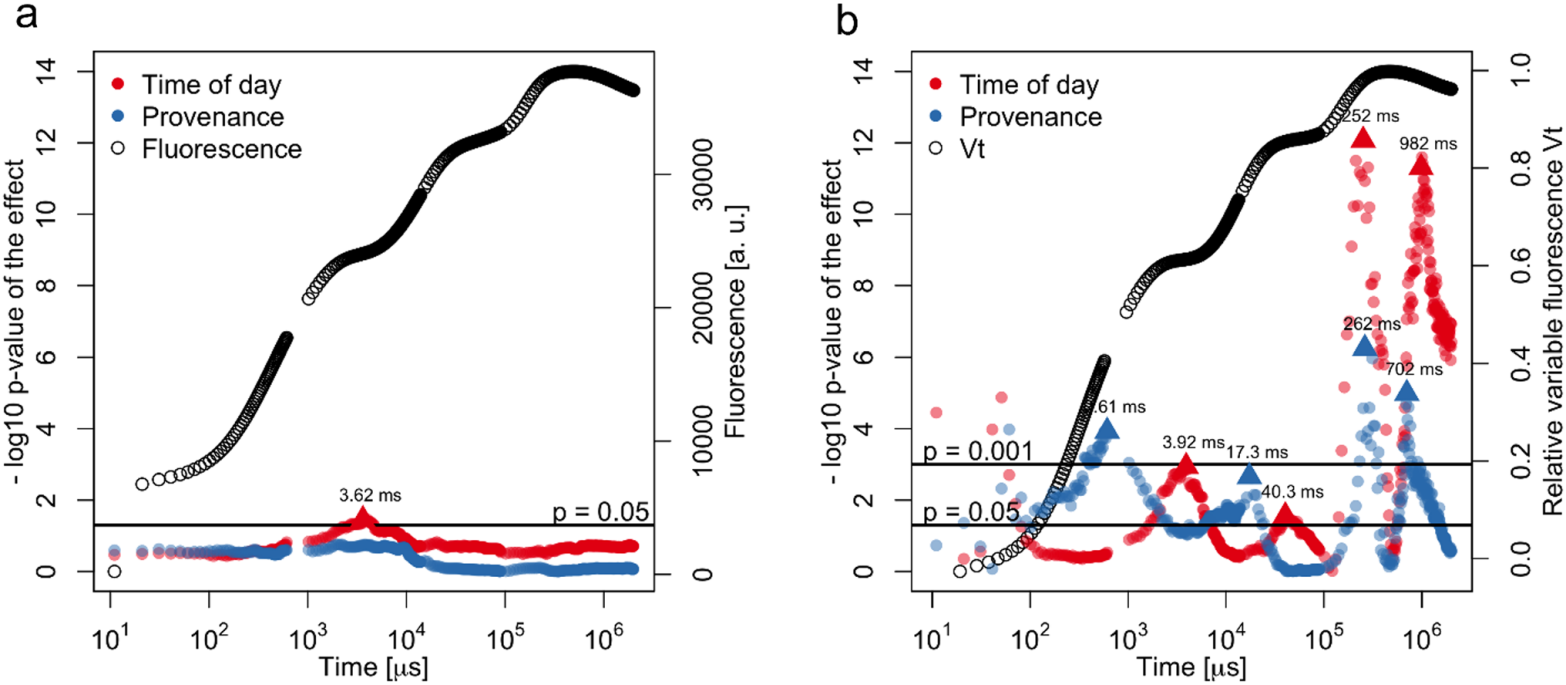
Significances of provenance and times of day factor as recorded by a nonparametric model applied along the OJIP and *V*_*t*_ transient for each recorded point. Significances along the OJIP transient (**a**) and *V*_*t*_ transient (**b**) plotted as the negative of its base 10 logarithm, with mean OJIP and *V*_*t*_ transients shown in black. Values above the reference lines were significant at indicated levels (*p* < 0.05, *p* < 0.001)

**Fig. 3:**
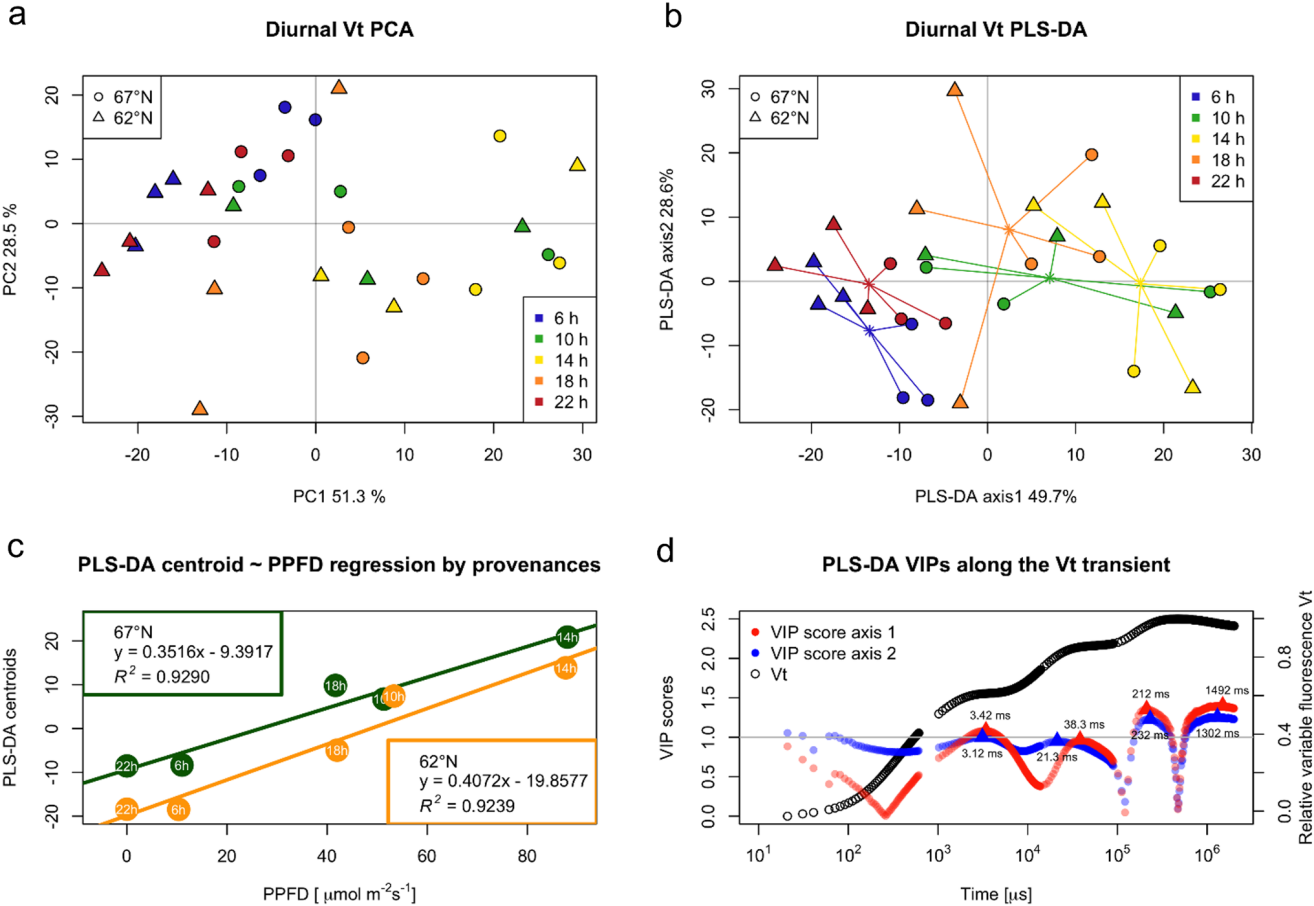
Multivariate statistical analysis of *V*_*t*_ values with emphasis on provenances and times of day. **a** Principal component analysis (PCA) plot with principal component 1 (explaining 51.3% of total variance) and principal component 2 (explaining 28.5% of total variance), the shape of the points denotes provenances, and colours denote times of day of sampling. **b** Partial least-squares discrimination analysis (PLSDA) 2D score plot with first two components for groups of times of day, centroids of each group plotted as asterisk. **c** Linear regression of PLS-DA centroids (position on the first component) and PPFD for respective provenances. When comparing provenance parameters, slope (*b*) difference was not statistically significant (*p* = 0.4743), intercept (*a*) difference was statistically significant (*p* = 0.0165). **d** Variable importance in projection (VIP) values for the first and second DA along the *V*_*t*_ transient (black). The threshold for meaningful VIP values is typically assumed to be 1 (i.e., larger than the average of squared VIP values)

The raw OJIP transients exhibited relatively large differences in fluorescence intensity among times of day and crown layers (Supplementary Fig. 3, and 7), but we used double-normalized *Vt* transients for the comparisons because of the focus on the kinetics of the transient (Kalaji et al. 2014a, b) (Fig. 1, 2, 3). We explored the use of the first and second derivatives to analyze when changes in the transients occur and to determine the locations of these landmarks. J and I steps were determined with the minima of the second derivative as in Tomek et al. (2001) and the other locations (1–5) with the minima and maxima of the first derivative (Fig. 1, Supplementary Fig. S4, Table 1). We tested for statistical differences among the occurrence times of the locations related to the time of day, provenances, and crown layers (Table 1). We then checked if the locations coincided with the times of significant differences according to the nonparametric repeated-measures model for provenance and times of day (Table 1a, Fig. 2).

**Table 1:** Statistical significance testing by the nonparametric repeated-measures model among the times of occurrence of the intermediate steps (Figs. 1 and 3) of the fluorescence transients (*p*-values < 0.05 in bold)

Parameters	Location (1)	Location (J-step)	Location (2)	Location	Location (I-step)	Location (4)	Location (5)	Location (P-step)	
(a)									
Provenance	0.086	0.857	0.893	0.129	0.294	0.273	0.127	0.075	
Time of day	0.219	**3.14E−05**	**9.75E−06**	**8.01E−06**	**0.002**	**0.0001**	**7.36E−08**	**9.35E−05**	
(b)									
Crown layers 7 July	**0.001**	0.058	0.054	0.358	0.314	**0.004**	**0.049**	**0.001**	
Crown layers 28 July	0.118	0.1	0.52	0.613	0.217	0.115	0.771	0.065	

(a) the time of day and provenance, (b) the crown layers, crown sides and sampling dates

### Diurnal variation in chlorophyll fluorescence kinetics

**Fig. 4:**
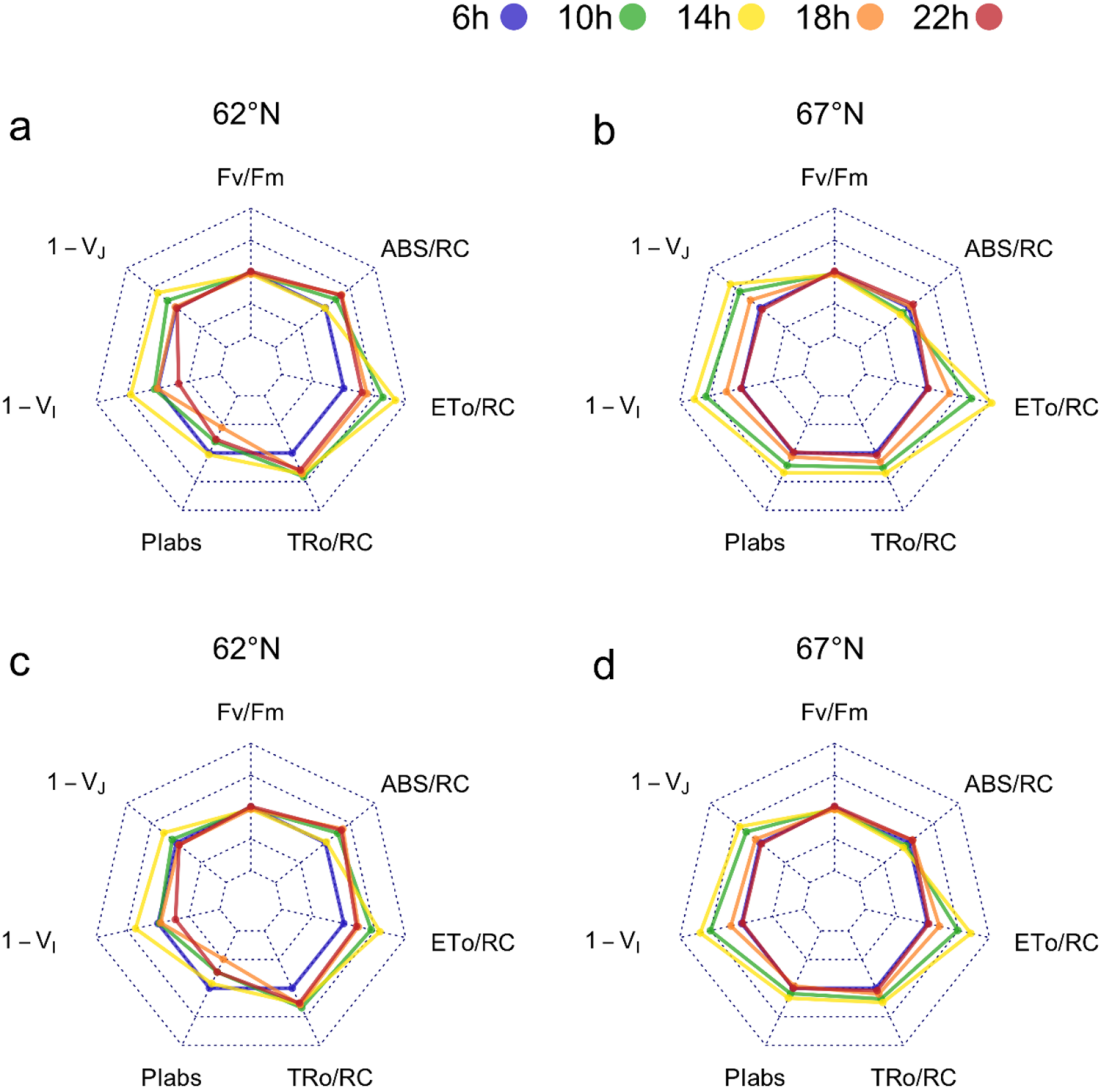
Diurnal variation of chlorophyll fluorescence parameters *F*_*v*_/*F*_*m*_, ABS/RC, ETo/RC, TRo/RC, PIabs, 1-*V*_*I*_, 1-*V*_*J*_, for silver birch provenances from northern (67°N, K1) and southern (62°N, V14) Finland at daytimes 6 h, 10 h, 14 h, 18 h, and 22 h. OJIP fluorescence parameters computed with the **A, B** time-adjusted JIP test and **C, D** traditional JIP test are compared. One concentric heptagon = 25% difference

Many of the fluorescence parameters computed with the traditional JIP test are dependent on the variable fluorescence at the J-step, and therefore, would be affected by the time adjustment with the second derivative of the transient proposed here. The comparison of the methods (Fig. 4, Table 1a and 2) revealed consistent patterns in the statistical differences between provenances and among the times of day in the results of the nonparametric repeated-measures test. For the time of day, the time-adjusted JIP test resulted in consistently smaller p-values for all the computed ChlF parameters (Table 2). Notably, 1-*V*_*I*_ differences were significant with the time-adjusted JIP test but not in the traditional JIP test with fixed times for the occurrence of J and I steps. By contrast, for provenance differences at different times of day, the time-adjusted JIP test resulted in higher p-values, except for ABS/RC. Here, the provenance difference for 1-*V*_*J*_ was significant in the traditional JIP test but not in the time-adjusted JIP test (Table 2). In Fig. 4, these differences are visualized in the wider spread of values for the time of day in the time-adjusted JIP test than in the traditional JIP test, but less clearly for provenance differences. The highest values for 1-*V*_*J*_, 1-*V*_*I*_ and ETo/RC were evident at 14 h in both provenances (Fig. 4).

**Table 2:** Nonparametric repeated-measures model for diurnal and provenance variation of chlorophyll fluorescence parameters in silver birch provenances from northern (67°N, K1) and southern (62°N, V14) Finland

Parameters	Time of day (adjusted)	Time of day (traditional)	Provenance (adjusted)	Provenance (traditional)	Daytime × provenance (adjusted)	Daytime × Provenance (traditional)
1-*V*_*J*_	**0.001**	**0.021**	0.077	**0.016**	0.771	0.762
1-*V*_*I*_	**0.026**	0.087	0.403	0.377	0.374	0.401
ABS/RC	0.255	0.351	**1.39E−04**	**4.53E−04**	0.476	0.606
TRo/RC	**0.010**	**0.047**	0.221	0.166	0.656	0.755
ETo/RC	**1.05E−04**	**1.20E−04**	0.713	0.614	0.546	0.573
PIabs	0.404	0.429	**0.003**	**0.001**	0.557	0.502
*F*_*v*_/*F*_*m*_	**8.73E−05**	**8.73E−05**	0.186	0.186	0.760	0.760

OJIP fluorescence parameters computed with the time-adjusted and traditional JIP tests are compared

*p*-values <0.05 in bold

For the southern provenance (62°N, V14), ChlF parameters exhibiting a linear relationship with photosynthetic photon flux density (PPFD, -μmol m^−2^ s^−1^) showed stronger linear regression values obtained by the time-adjusted JIP test than the traditional JIP test (Table 3, Supplementary Fig. S5 a,b). For the northern provenance (67°N, K1), ABS/RC, TRo/RC and PIabs showed no linear relationship with PPFD, but for the other parameters, the time-adjusted JIP test approach resulted in higher *R*^2^ values than the traditional JIP test.

**Table 3:** *R*^*2*^ values for the regression matrix of photosynthetic photon flux density (PPFD, μmol m^−2^ s^−1^) at different times of day for chlorophyll fluorescence parameters

Parameter	(62°N, K1)		(67°,V14)	
	Adjusted	Traditional	Adjusted	Traditional
1-*V*_*J*_	0.958	0.912	0.814	0.672
1-*V*_*I*_	0.910	0.892	0.802	0.704
ABS/RC	0.714	0.482	− 0.146	− 0.213
TRo/RC	0.922	0.873	0.105	− 0.024
ETo/RC	0.947	0.904	0.705	0.684
PIabs	0.904	0.530	− 0.256	− 0.329

OJIP fluorescence parameters computed with the time-adjusted and traditional JIP tests are compared

Diurnal variation in fluorescence transients was studied five times a day (6 h, 10 h, 14 h, 18 h, and 22 h) with northern (67°N, K1) and southern (62°N, V14) provenances of Finnish silver birch (Fig. 1 and 2, Supplementary Fig. S6). The provenances exhibited some differences along the transient, mainly at P and after the J-step (Fig. 1a), but none of the differences in the time of occurrence for the locations was significant (Table 1). Diurnal curves in the Vt transient varied considerably after the inflection point of the O–J rise at around 400 µs (location 1), visualized most clearly in the first derivative curve at location 5 (Fig. 1b). Except for location 1, all the landmarks showed significant differences in occurrence times (Table 1). The J-step occurred at about the conventional 2 ms, but the I-step occurred earlier than the fixed 30 ms of the traditional JIP test. The J and I steps occurred earlier when irradiance was most intense (at 14 h) and latest at 6 and 22 h when light intensity was lowest (Fig. 1b). *V*_*t*_ curves and derivatives of the silver birch leaves at 6 h were similar to those at 22 h (Fig. 1).

According to the repeated-measures model, the times of day or provenances did not differ along the raw OJIP transient except for the diurnal difference at 3.62 ms after the J step, which coincides with location 2 determined by the first derivative (Fig. 1b and 2a). Along the Vt transient, diurnal differences were found at 3.92 ms after the J-step (location 2), at 40.3 ms (location 4), and around the P-step at 252 ms (location 5) and at 982 ms (Fig. 1b and 2b). Provenance differences were found at 0.61 ms (second derivative minimum, Supplementary Fig. S4), 17.3 ms (location 3), 262 ms (location 5), and 702 ms after the P-step (Fig. 1b and 2b).

**Fig. 5:**
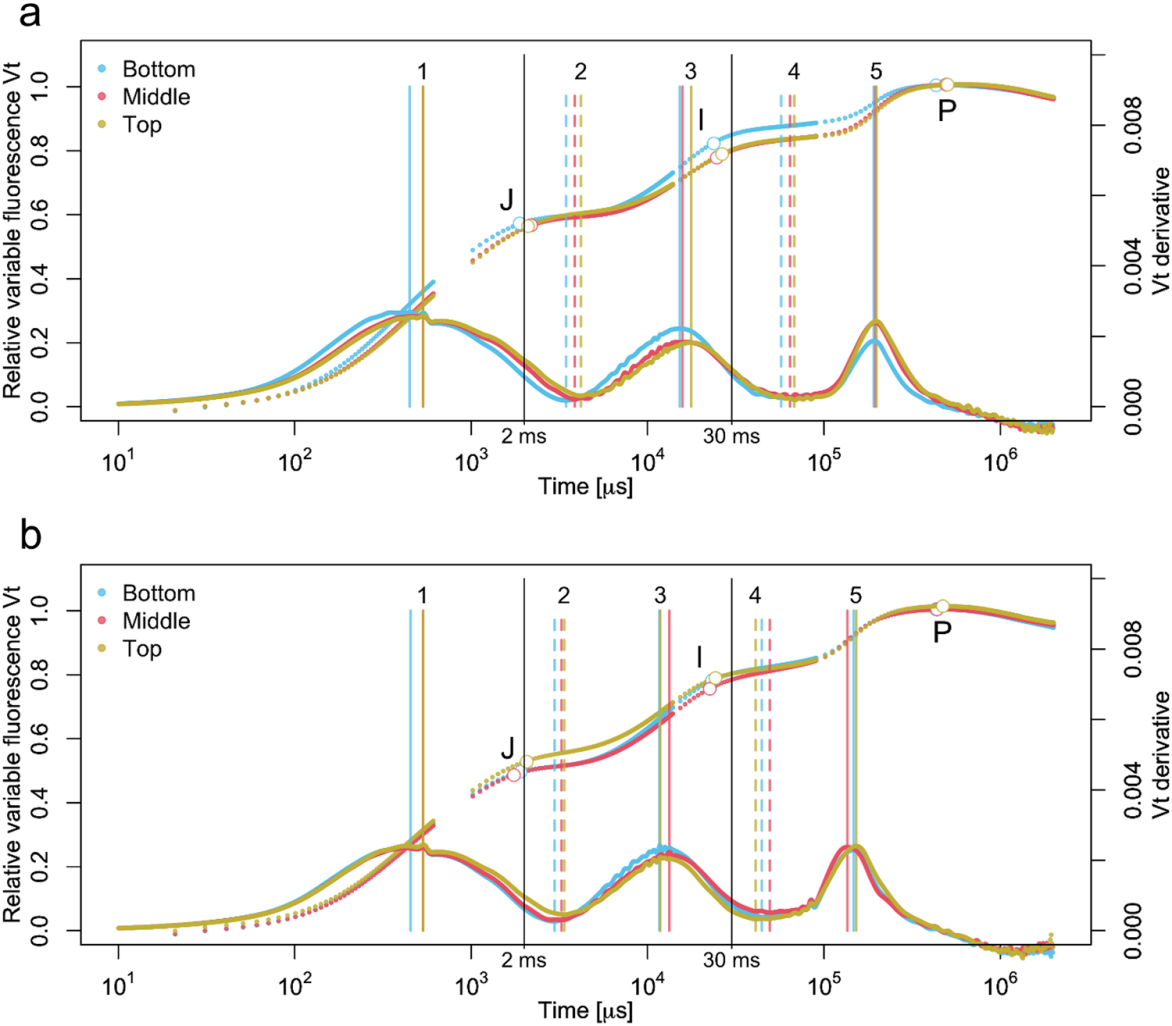
Curves of relative variable fluorescence (*V*_*t*_) transient of within-crown variation in silver birch for different crown layers (bottom, middle, top) for two sampling days, July 7th (**a**) and July 28th (**b**). Upper curves show normalized variable fluorescence (*V*_*t*_) transient for different crown layers and lower curves show 1st derivative of Vt transient in both (**a** and **b**). Positions of the J and I steps for the time-adjusted JIP analysis are marked as small circles in the curves, while positions of the J (2 ms) and I (30 ms) and other intermediate steps (locations 1–5) for the traditional JIP test are shown as vertical lines. Positions of plateaus and inflection points are displayed as vertical lines

We used PCA on fluorescence intensity data along the whole Vt transient to explore the differences between provenances in relation to diurnal variation. The morning and evening measurements (6 h and 22 h) formed a loose cluster (Fig. 5a), but there were no obvious differences between provenances. We further examined the fluorescence data for separating different times of day with discriminant analysis (PLS-DA) (Fig. 5b). The morning and evening measurements (6 h and 22 h) clustered together, and the time of day measurements formed a separate overlapping grouping. Moreover, the provenances were clearly separated from each other in the 6 and 22 h measurements but not at time of day measurements as shown also in the radar plots of the JIP test parameters (Fig. 5b, and Supplementary Fig. S6). The first DA axis appeared to be related to differences in light intensity, increasing from left to right. The location of group centroids in the first DA axis showed a strong linear relationship with photosynthetic photon flux density with both provenances (Fig. 5c). The regression parameters for provenance showed a statistically significant difference (*p* = 0.0165) for the intercept (*a*), but in slope (*b*), they did not differ (*p* = 0.4743). The variable importance in projection (VIP) values for the first and second DA axis (Fig. 5d) showed values higher than 1 at the I–P phase and after the P step, similar to the significance of the diurnal difference in the repeated-measures model (Fig. 2b).

### Within-crown variation in chlorophyll fluorescence kinetics

Within-crown variation was studied for differences among crown layers, cardinal directions and on two sampling dates. Crown layer differences were detectable around the J and I plateaus of the *V*_*t*_ transient (Fig. 3) and raw OJIP fluorescence transient (Supplementary Fig. S7). Among the crown layers, significant shifts can be seen at locations determined by the derivatives for different sampling dates; July 7 and 28 (Fig. 3). These locations differed significantly at locations 1, 4, 5 and at the P-step on July 7, with no significant differences observed on July 28. (Table 1b). The occurrence times of landmark events along the OJIP transient occurred faster at the bottom layer on July 7, and on July 28, faster occurrence times of OJIP landmarks was also observed from locations 1 to 3.

Parameters related to the apparent antenna size, trapping flux, and electron transport per reaction centre (ABS/RC, ET_0_/RC and TR_0_/RC,) were higher at the bottom layer than at upper layers, and these differences were more pronounced using the time adjustment in JIP test. There were no obvious differences among the cardinal directions of the tree crown, except around the P step (Supplementary Fig. S7). The dataset analysed separately for the two measuring days shows that the time-adjusted method worked better also for the within-tree variation analyses than the traditional OJIP method. (Table 4, Fig. 3, and Supplementary Fig. S8).

**Table 4:** Linear mixed model for crown layer variation of chlorophyll fluorescence parameters in Finnish silver birch V14 (62°N, southern provenance) (*p*-values < 0.05 in bold)

Parameters	July 7th (adjusted)	July 7th (traditional)	July 28th (adjusted)	July 28th (traditional)
1-*V*_*J*_	0.609	0.230	0.161	0.213
1-*V*_*I*_	**0.003**	**0.006**	0.394	0.231
ABS/RC	**0.002**	**0.009**	0.672	0.710
TRo/RC	** < 0.0001**	** < 0.0001**	**0.029**	0.053
ETo/RC	**0.005**	**0.039**	**0.034**	**0.031**
PIabs	0.532	0.527	0.354	0.393

OJIP fluorescence parameters computed with the time-adjusted and traditional JIP tests are compared for sampling dates; July 7th and July 28th

## Discussion

In this study, we used a combination of the first and second derivatives of the fast ChlF transient to determine the landmark locations along the OJIP curve. The OJIP curve is treated as a graph of a continuous function where the change and the rate of change of fluorescence intensity determine the shape of the curve. Thus, derivatives can be used to localize the OJIP curve landmarks, such as the inflection points where the concavity of the curve changes from upward to downward or vice versa. To use the derivatives, it is necessary to approximate a continuous curve from the point measurements of the device. This can be achieved using piecewise low-degree polynomials with splines or higher-degree polynomial fitting, as in Tomek et al. (2001). In previous studies, derivatives have been used only for the determination of the occurrence of J and I steps (Tomek et al. 2001; Xia et al. 2019). Here, we have used derivative-based OJIP transient analysis as a tool to visualize and quantify the extent of time shifts occurring in OJIP fluorescence curves, especially at J and I steps and in addition, we were able to point out other landmark locations of the OJIP transient obtained using the OJIP curve inflection points. We compared these results with the traditional JIP-test that uses fixed times.

### Diurnal variation in chlorophyll fluorescence kinetics

The occurrence times of the J and I steps determined using derivatives showed variation between provenances and among different times of the day. Differences in plant physiological status and experimental conditions lead to significant changes in the shape of the OJIP induction curve, shifting the position of J and I steps either earlier or later (Tomek et al. 2001; Xia et al. 2019; Guo et al. 2020). Variations in earlier occurrence times for the I and J steps have been reported among oak seedlings from different provenances, especially as the I-step occurred significantly earlier in the provenance that was the most affected by drought stress (Bantis et al. 2020). During the day, our results showed the J and I steps occurred earlier in high light intensity and later in low light conditions. This supports previous findings that photosynthesis is faster when light intensity increases, and accelerated reaction rates lead to earlier J and I transition (Qiang and Richmond 1996; Johkan et al. 2012; Stirbet and Govindjee 2012).

The analysis of large ChlF datasets and detection of hidden information in ChlF data is attainable by utilizing secondary data processing such as the principal component analysis (PCA) (Pollastrini et al. 2016; Kalaji et al. 2018) or artificial neural networks (ANNs) (Kohonen 2012; Kalaji et al. 2017). Here, the PLS-DA revealed provenance differences under low light conditions, especially at 22 h, suggesting a difference in the adjustment of photosynthetic activity to varying availability of light. This suggests that the photosynthetic system in northern trees remains active in low light levels, possibly overnight, as they are genetically adapted to the 24 h daylight conditions at their place of origin during the summer months in Finland, as was found in studies on gas exchange and photosynthetic rates for the same birch provenances (Tenkanen et al. 2021, 2023). The Northern Kittilä (67° N) provenance has been shown to have higher maximum quantum efficiency (*F*_*v*_/*F*_*m*_) than a southern provenance in uniform conditions (Tenkanen et al. 2021). The lower values of apparent antenna size of active PSII (ABS/RC) in the northern trees, but with an overall better performance index compared to the southern trees, suggests that the antenna size or absorption of photons per reaction center may not be the primary determinant of better overall photosynthetic efficiency. These are possibly more reliant on trapping probability and efficiency in electron transport, as shown in the higher photosynthetic efficiency of the northern trees under lower irradiance.

The diurnal differences in both provenances were further examined by comparing variations in light intensity (PPFD) with ChlF parameters. Following the diurnal trend of PPFD, ChlF parameters at 22 h often returned to values close to the first measurement taken in the early morning, as reported for grassland species (Digrado et al. 2018). *F*_*v*_/*F*_*m*_ showed a negative relationship with PPFD, which is in line with the well-known midday depression of *F*_*v*_/*F*_*m*_ values (Adams and Demmig-Adams 2004; Desotgiu et al. 2012; Pellegrini et al. 2015). During the day, increased light intensity could enhance heat damage of PSII, especially when the light stress combines with increased temperature and transpiration rate (Srivastava and Strasser 1996). 1-*V*_*J*_, 1-*V*_*I*_, and ET_0_/RC showed a positive relationship with PPFD, and the coefficients of determination of the regression were higher with the time-adjusted JIP test approach. Khan et al. (2020) showed that, together with temperature, PAR is a dominant environmental factor greatly influencing the ChlF-derived parameters, especially the *F*_*v*_/*F*_*m*_, the J–I phase, and the I–P phase of the OJIP transient, but not influencing the ABS/RC. In our study, regression of PPFD with ABS/RC, TR_0_/RC and PIabs indicated a linear relationship with the southern provenance (62°N, V14) but not with the northern provenance (67°N, K1).

### Within-tree variation in chlorophyll fluorescence kinetics

Light intensity appeared to have opposite effects on the appearance of the J and I steps in the diurnal variation and within-crown experiments. In the diurnal variation experiment, the J and I steps occurred earlier in high than in lower light intensity. However, among the crown layers, the J and I steps occurred earliest at the bottom layer with the lowest light intensity and latest at the top layer. Our 5-year-old silver birch trees were 4–6 m in height and, due to small spacing between trees, had a dense canopy allowing less light penetration to lower parts of the trees. Leaves at the bottom of the crown had a higher absolute fluorescence intensity along the whole V_t_ transient than leaves at the upper layers, apparently because they were acclimated to shade conditions and were less capable of quenching the saturating light pulse photosynthetically.

In comparison to the bottom crown layer, the observed lower values of *F*_*v*_/*F*_*m*_ at the top crown caused by a higher level of irradiance has been reported for forest trees (Pollastrini et al. 2017), and may reveal either a process of photodamage in PSII or the onset of a photoprotection mechanism to deal with the excess level of light energy (Demmig and Björkman 1987). Additionally, parameters related to the electron transport chain and light-harvesting antenna size (ABS/RC, TR_0_/RC, and ET_0_/RC) were affected by light intensity and were higher at the bottom crown than at the top crown. Top crown leaves possess a reduced electron trapping capacity but have an increased capacity to reduce the final acceptors of electrons beyond PSI (Cascio et al. 2010; Desotgiu et al. 2012).

Increased 1-*V*_*I*_ values observed at the top and middle crown layer could indicate a greater relative size of the final electron acceptor pool in photosystem I. This phase primarily depends on the relative composition of PSI; electron flow via PSI and a transient obstruction in the flow of electrons on the acceptor side of PSI (Desotgiu et al. 2012).

For the ChlF parameters that showed significant differences in both the experimental days, the significant difference values obtained by the time-adjusted JIP test were better than the traditional JIP test. Results obtained for both experimental days differed considerably possibly due to changes in daily environmental conditions, effect of shading and PPFD variation among individuals measured, seasonal progression and leaf age (Mattila et al. 2021). Variations in ChlF kinetics resulting from leaf morphological changes may also occur during the growing season (Swoczyna et al. 2022).

### The use of derivatives and consideration of the OJIP curve time domain

Derivatives manifest the rate of change in fluorescence intensity and are valuable for quantitative assessment of visually undetectable amplitude and half-time of the OJIP phases along the time axis of the fluorescence transient (Boisvert et al. 2006; Khan et al. 2020). Derivatives also provide added value for interpreting the behaviour of the OJIP curve and ChlF signals under varying light quality (Xia et al. 2019). Our use of the first derivative transformation and determination of the occurrence time of J and I steps with the second derivative enabled visualization and localization of significant time of day, provenance-related, and crown layer differences at potentially important steps, landmarks, or phases along the OJIP transient. The second derivative transformation (Supplementary Fig. S4) revealed additional local minima and maxima along the OJIP transient that may have further potential for fluorescence analysis. Although it is reasonable to assume that these may have a molecular basis in the dynamics of the system, for the demonstration of the method, consideration of these was not deemed necessary.

Statistical differences among the occurrence of landmark locations for the time of day and provenances coincided with significance peaks determined by the nonparametric repeated-measures model along the OJIP curve. Even though the number of replicates in this study was low, the tests showed consistent results. The application of time adjustment in the JIP test also increased the statistically significant differences among the times of day and among the crown layers in most calculated ChlF parameters that are influenced by the occurrence times of J and I. Furthermore, our results indicate the importance of the kinetics of the OJIP curve, as in the time domain, the times of day differed significantly at the early phases of the OJIP curve (before 4 ms), but for the fluorescence intensities, at the end of the *V*_*t*_ transient (after 100 ms). Our approach gives an opportunity to analyze not only the fluorescence intensity variation along the OJIP transient but also the potential significance of the occurrence times of the intermediate steps along the transient’s time domain.

## Conclusion

The derivative-based analysis, presented as an alternative approach for analyzing ChlF kinetics data showed important light-induced variations in the OJIP transient. The most significant time of day or provenance-related differences in fluorescence intensity were noticeable at the landmark locations determined by our use of derivatives. Results on diurnal and within-crown variation suggest that the time-adjusted JIP test approach provides additional value for ChlF data analysis, particularly if the experimental questions are related to variations in ambient light intensity or other environmental factors that could cause time shifts in the occurrence of landmark events in the fluorescence transient. Taken together, our results emphasize the potential relevance of considering the time domain in the analysis of the fast ChlF induction.

### Supplementary Information

Below is the link to the electronic supplementary material.

Supplementary file1 (RMD 16 KB)

Supplementary file2 (DOCX 4257 KB)

## Data Availability

The data are available upon reasonable request from the corresponding author.
