# Correction to: Derivative-based time-adjusted analysis of diurnal and within-tree variation in the OJIP fluorescence transient of silver birch

**DOI:** 10.1007/s11120-023-01050-w

**Published:** 2023-09-21

**Authors:** Olusegun Olaitan Akinyemi, Jaroslav Čepl, Sarita Keski-Saari, Ivana Tomášková, Jan Stejskal, Sari Kontunen-Soppela, Markku Keinänen

**Affiliations:** 1 Department of Environmental and Biological Sciences, University of Eastern Finland, Yliopistokatu 7, P.O. Box 111, 80101 Joensuu, Finland; 2 Department of Genetics and Physiology of Forest Trees, Czech University of Life Sciences Prague, Kamýcká 129, 165 00 Prague 6, Czech Republic; 3 Center for Photonics Sciences, Yliopistokatu 7, P.O. Box 111, 80101 Joensuu, Finland

**Photosynthesis Research (2023) 157:133–146** 10.1007/s11120-023-01033-x

The original article has been corrected: Figure legends 3 and 5 had been inversed and have now been correctly rearranged.

