# Supplementary material for: Derivative-based time-adjusted analysis of diurnal and within-tree variation in the OJIP fluorescence transient of silver birch
Source: Photosynth Res. 2023 Jun 29;157(2-3):133–46. doi: 10.1007/s11120-023-01033-x (PMC10485093; doi:10.1007/s11120-023-01033-x)
Supplement: Supplementary file 2 — Supplementary file2 (DOCX 4257 KB) [file 11120_2023_1033_MOESM2_ESM.docx]

**Supporting Information**
*Olusegun Olaitan Akinyemi^*^, Jaroslav Čepl, Sarita Keski-Saari, Ivana Tomášková, Jan Stejskal, Sari Kontunen-Soppela^# 1^, and Markku Keinänen^# 1,3^*

**Derivative-based time-adjusted analysis of diurnal and within-tree variation in the**

**OJIP fluorescence transient of silver birch**

## * Corresponding author: Olusegun Olaitan Akinyemi: [olusegun.akinyemi@uef.fi](mailto:olusegun.akinyemi@uef.fi)

## # Authors contributed equally

**The following Supporting Information is available for this article:**

**Supplementary Table S1:** Average diurnal PPFD (μmol m^-^² s^-1^) values

**Supplementary Table S2:** Definitions and derivations of measured ChlF parameters

**Supplementary Figure S3:** Diurnal OJIP transient and its first-order derivatives.

**Supplementary Figure S4:** Vt transient and its first and second-order derivatives

**Supplementary Figure S5 a,b:** Regression matrix of PPFD and ChlF parameters. Time-adjusted JIP and traditional JIP tests are compared.

**Supplementary Figure S6:** Diurnal variation of ChlF parameters. Time-adjusted JIP and traditional JIP tests are compared.

**Supplementary Figure S7:** Within-crown OJIP transient and its first-order derivatives.

**Supplementary Figure S8:** Within-crown and crown side variation of ChlF parameters in silver birch. Time-adjusted JIP and traditional JIP tests are compared.

**Supplementary Table S1** Average value of photosynthetic photon flux density (PPFD, μmol m^-^² s^-1^) for each sampling hour on July 16, 2015, at dawn (6h), morning (10h), midday (14h), evening (18h), and night: (22h).

| **Time** | **Genotype** | **PPFD** [μmol m^-^² s^-1^] |
| --- | --- | --- |
| 6 | V14 (62°N) | 10.3 |
| 10 | V14 (62°N) | 53.3 |
| 14 | V14 (62°N) | 87.7 |
| 18 | V14 (62°N) | 42.0 |
| 22 | V14 (62°N) | 0.0 |
| 6 | K1(67°N) | 11.0 |
| 10 | K1(67°N) | 51.3 |
| 14 | K1(67°N) | 88.0 |
| 18 | K1(67°N) | 41.7 |
| 22 | K1(67°N) | 0.0 |

**Supplementary Table S2** Definitions and derivations of measured chlorophyll fluorescence parameters (Bussotti et al. 2010; Stirbet and Govindjee 2011; Sipka et al. 2021).

F_t_ Fluorescence intensity from a dark-adapted leaf at time t

F_0_ Minimal fluorescence intensity from a dark-adapted leaf

F_K_ Fluorescence intensity at K-step

F_J_ Fluorescence intensity at J-step

F_I_ Fluorescence intensity at I-step

F_M_ Maximal fluorescence intensity

F_V_ Maximal variable fluorescence from a dark-adapted leaf F_V_ = F_M_ - F_0_

M_0_ Approximated initial slope of the fluorescence transient 4(F_K_ - F_0_) / (F_M_ - F_0_)

V_J_ Relative variable fluorescence intensity at J-step V_J_ = (F_J_- F_0_) / (F_M_ - F_0_)

V_I_ Relative variable fluorescence intensity at I-step V_I_ = (F_I_- F_0_) / (F_M_ - F_0_)

V_t_ Relative variable fluorescence at the time t. V_t_ = (F_t_ **-** F_0_) / (F_M_ **-** F_0_)

Fv/F_M_ Trapping probability or maximum quantum yield of primary photochemistry of a dark-adapted leaf. This shows the probability that the Photosystem II reaction centre will trap an absorbed photon.
Fv/Fm is valuable for monitoring the functioning of the Photosystem II.

1-V_J_ Probability with which a PSII trapped electron is transferred beyond Q_A_

1-V_I_ Probability with which a PSII trapped electron is transferred until PSI acceptors

ABS/RC Apparent antenna size of an active PSII. ABS/RC = M_0_ × (1/ V_J_) × [1 - (F_0_/ F_M_)]

TR_0_/RC Trapping flux leading to QA reduction per reaction centre. TR_0_/RC = M_0_ × (1/ V_J_)

ET_0_/RC Electron transport in an active reaction centre. ET_0_/RC = (*M_0_/V_J_*) × (1-V_J_)

PI_abs_ Performance Index (potential) for energy conservation from photons absorbed by Photosystem II to reducing intersystem electron acceptors. PI_abs_ = RC/ABS [ Fv/F_M_ / (1- Fv/F_M_)] [1- V_J_ / 1- (1-V_J_)]


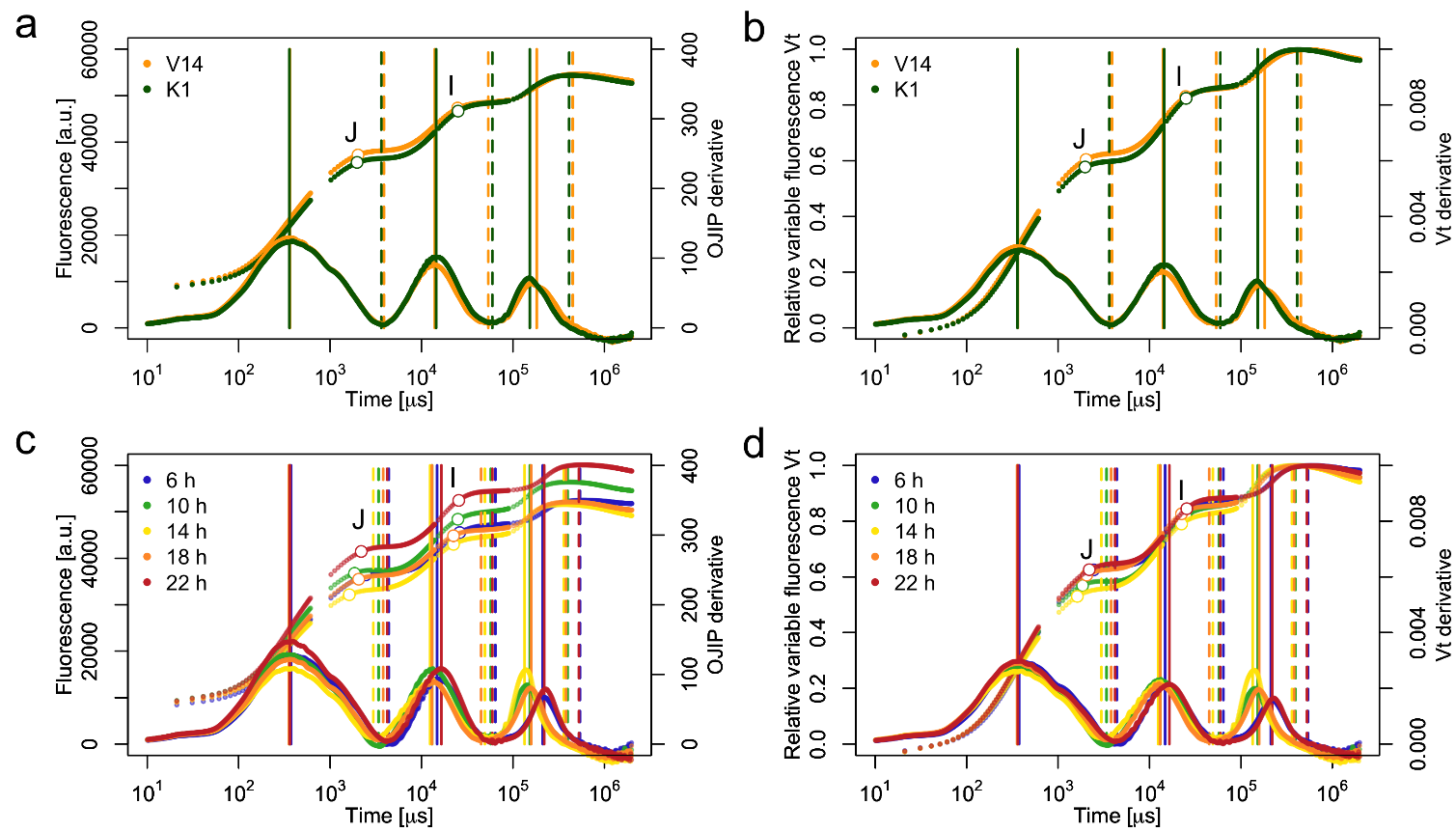


**Supplementary Fig. S3** Curves of OJIP fluorescence transient of silver birch provenances from northern (67°N, K1) and southern (62°N, V14) Finland measured at five times of day. Upper curves show OJIP transient for different provenances (a) and times of day (c). Lower curves show the 1^st^ derivative of transient. Mean curve of each provenance (a) or time of day (c) are displayed, with vertical lines showing the positions of plateaus and inflection points for each provenance or time of day separately. Positions of the J and I steps for the time-adjusted JIP analysis are marked as small circles in the curves, while positions of the J (2 ms) and I (30 ms) steps for the traditional JIP method are shown. The combined OJIP curve of the two provenances is shown for the diurnal variation.


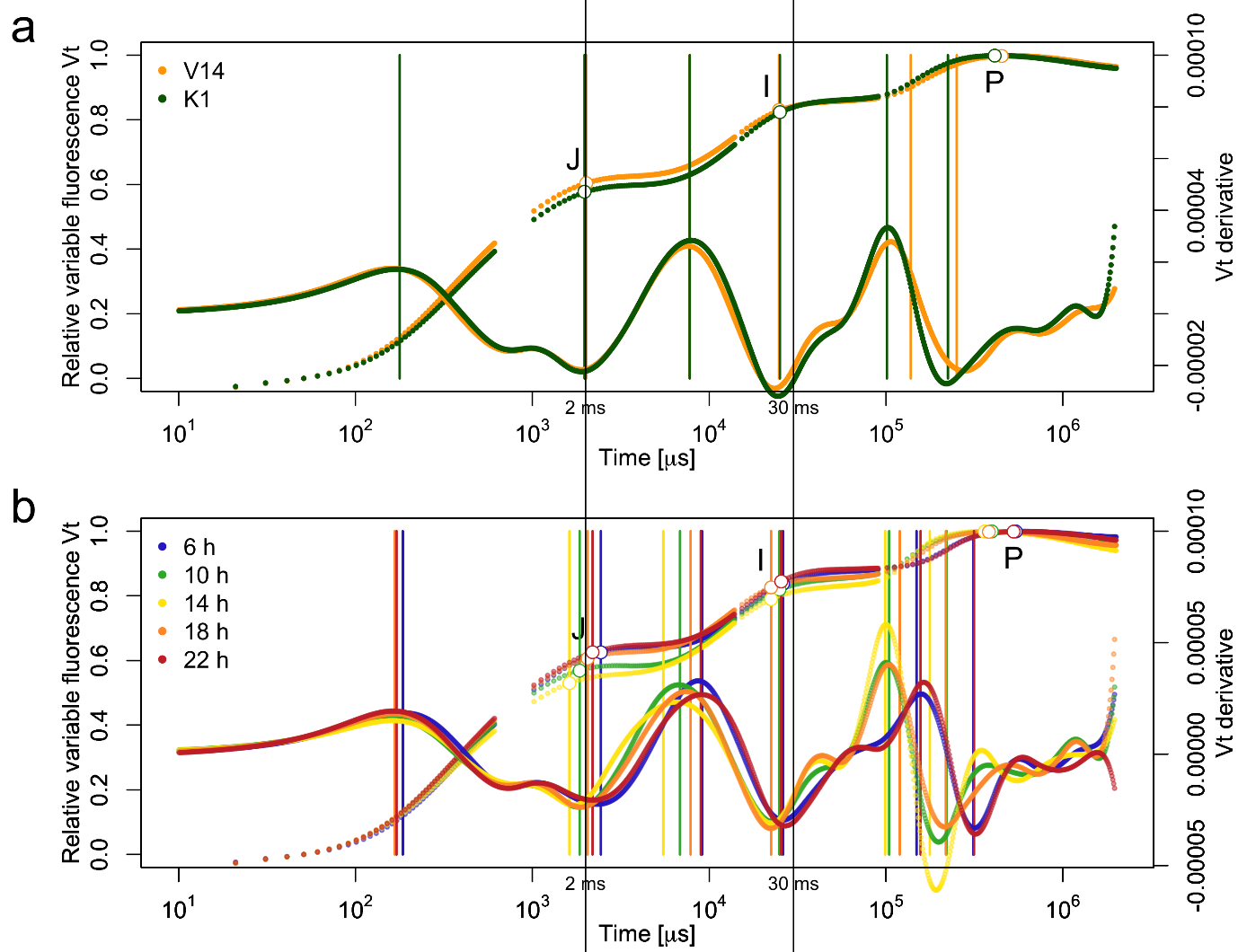


**Supplementary Fig. S4** Curves of relative variable fluorescence (Vt) transient of silver birch provenances from northern (67°N, K1) and southern (62°N, V14) Finland measured at five times of day. Upper curves show Vt transient for different provenances (a) and times of day (b). Lower curves show the 2^nd^ derivative of Vt transient. Mean curve of each provenance (a) or time of day (b) are displayed, with vertical lines showing the positions of plateaus and inflection points () for each provenance or time of day separately. Positions of the J and I steps, and for the time-adjusted JIP analysis are marked as small circles in the curves, while positions of the J (2 ms) and I (30 ms) steps for the traditional JIP method are shown. The combined OJIP curve of the two provenances is shown for the diurnal variation.


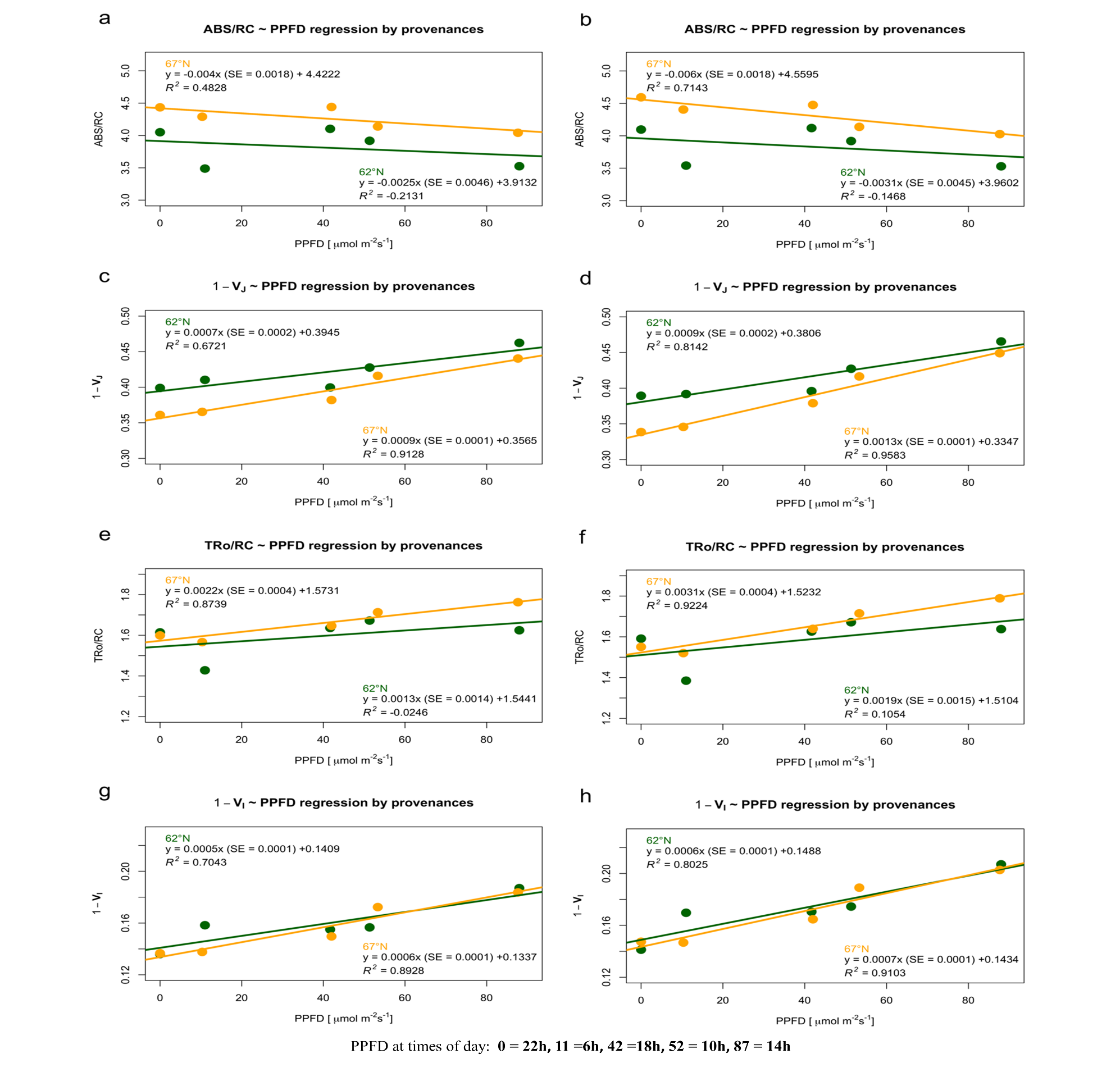
 **Supplementary Fig. S5a** Regression matrix of Photosynthetic photon flux density (PPFD, μmol m^-^² s^-1^) at different times of day for values of ChlF parameters. Raw OJIP fluorescence indices computed with the traditional JIP (left) and time-adjusted JIP (right) tests are compared.


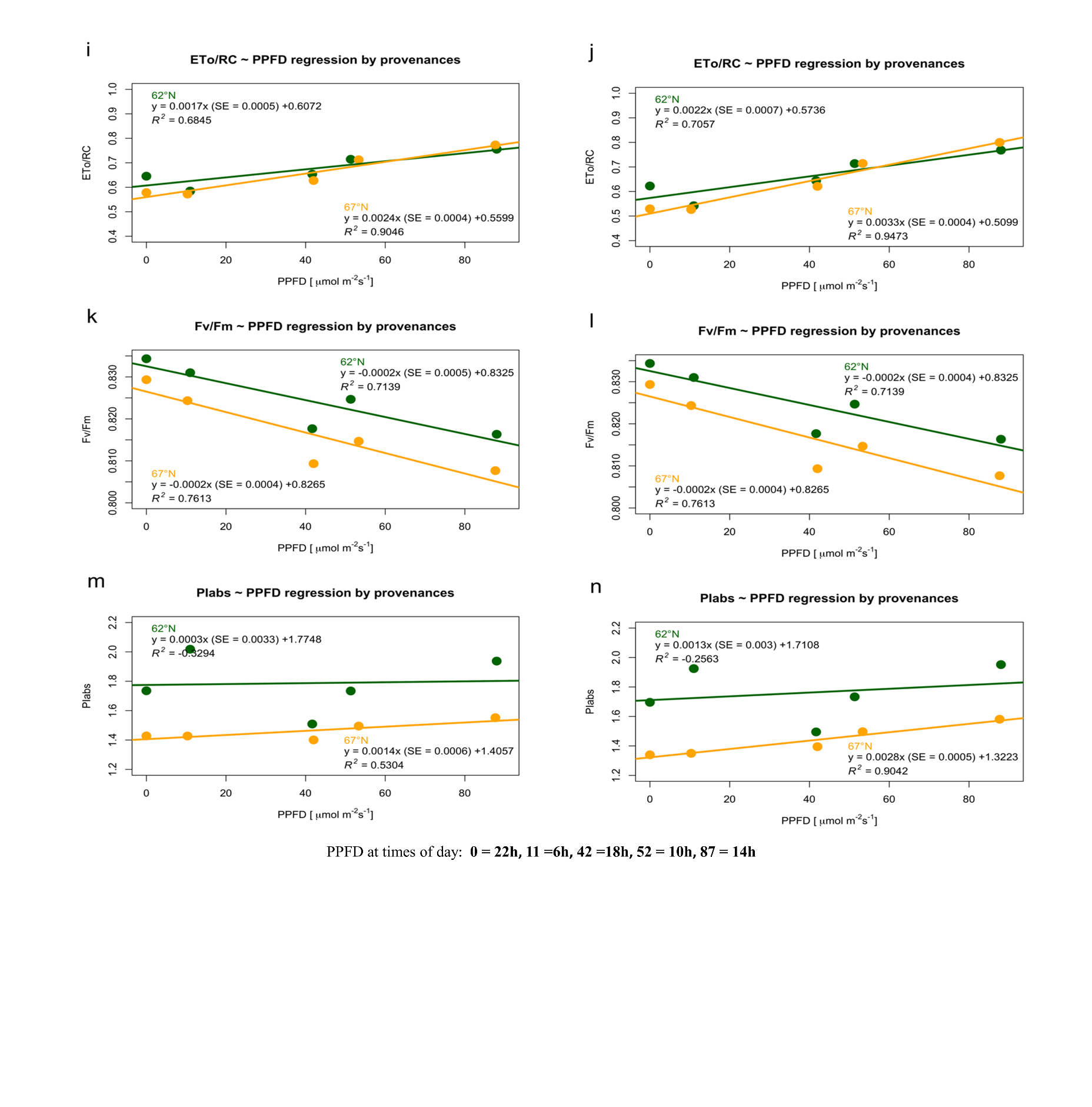


**Supplementary Fig. S5b** Regression matrix of Photosynthetic photon flux density (PPFD, μmol m^-^² s^-1^) at different times of day for values of ChlF parameters. Raw OJIP fluorescence indices computed with the traditional JIP (left) and time-adjusted JIP (right) tests are compared.


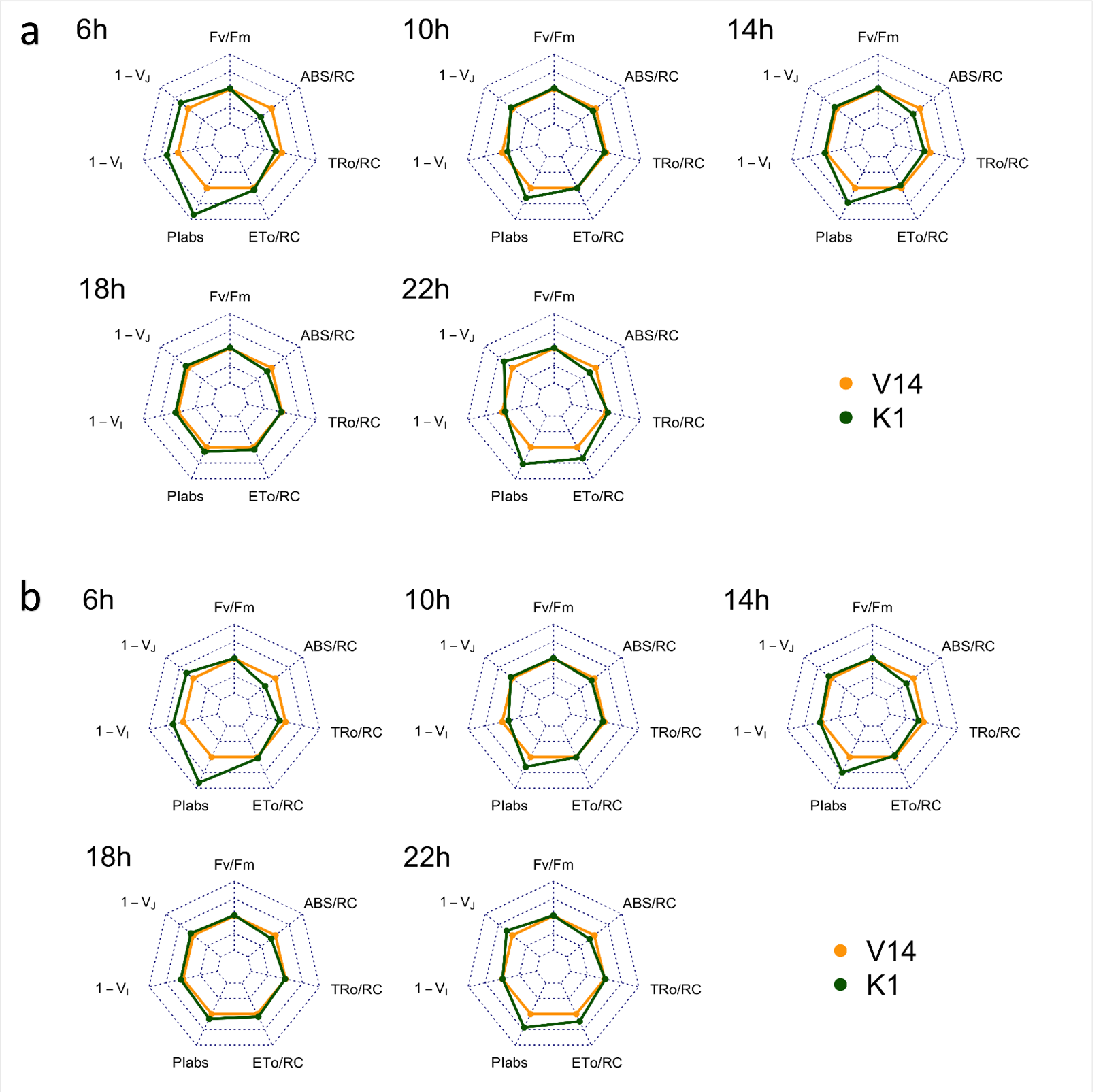


**Supplementary Fig. S6** Diurnal variation of chlorophyll fluorescence parameters Fv/Fm, ABS/RC, TRo/RC, ETo/RC, PIabs, 1-V_I_, 1-V_J_, for Finnish silver birch K1 (67°N, northern provenance) and V14 (62°N, southern provenance) at daytimes 6h, 10h, 14h, 18h, and 22h.
Raw OJIP fluorescence indices computed with the **(a)** time-adjusted JIP method and **(b)** traditional JIP method are compared. One concentric heptagon = 25% difference.


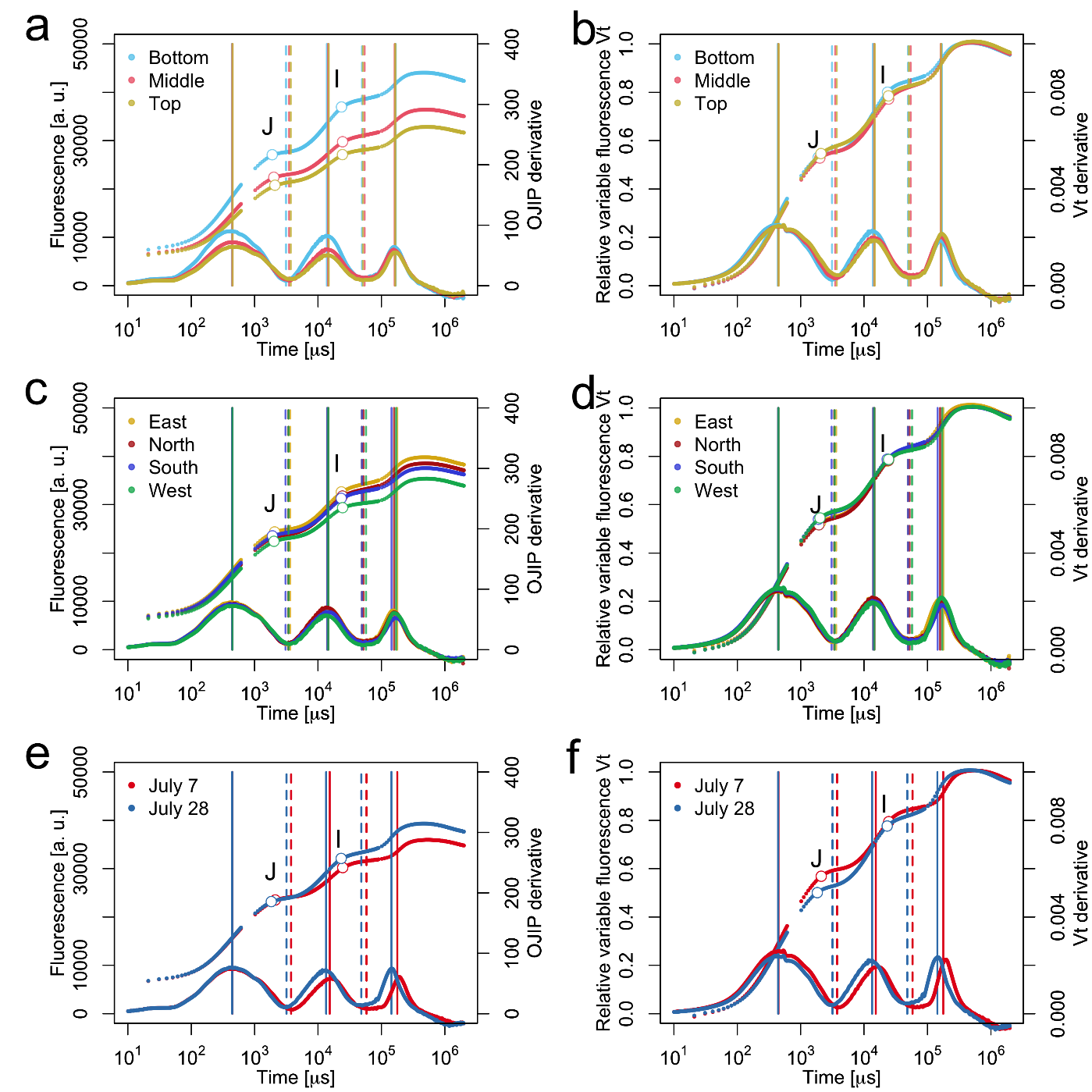


**Supplementary Fig. S7** Curves of OJIP fluorescence transient of within-crown variation in silver birch for different crown heights (bottom, middle, top), sides and sampling date. Nonparametric repeated-measures model significances for each point of OJIP transient plotted as the negative of its base 10 logarithm, mean fluorescence transient in the background, below it: OJIP transient with mean curves & its derivatives for crown heights, sides and sampling date. Positions of the J and I steps for the time-adjusted JIP analysis are marked as small circles in the curves, while positions of the J (2 ms) and I (30 ms) steps for the traditional JIP method are shown. Upper curves show OJIP fluorescence transient for different crown heights (a), sides (c), and sampling dates (e). Lower curves show 1^st^ derivative of OJIP transient, the mean curve for crown heights (a), sides (c) and sampling date (e). Positions of plateau and inflection points are displayed as vertical lines.


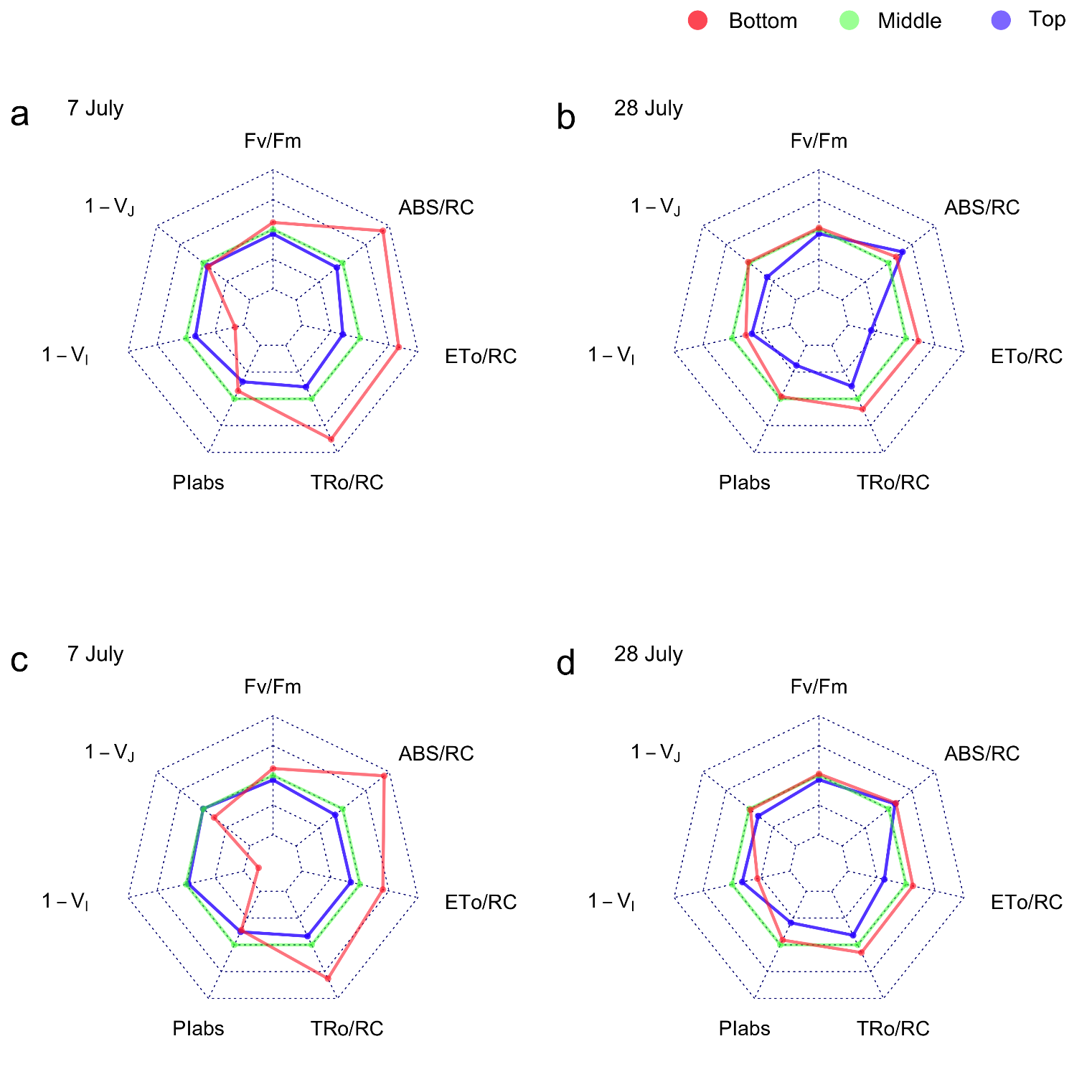


**Supplementary Fig. S8** Within-crown variation of chlorophyll fluorescence parameters, Fv/Fm, ABS/RC, ETo/RC, TRo/RC, PIabs, 1-V_I_, 1-V_J_, for different crown heights on 7th July (a, c), and 28th July (b, d) in silver birch. Raw OJIP fluorescence indices computed with the **(a, c)** time-adjusted JIP method and **(b, d)** traditional JIP method are compared. One concentric heptagon = 25% difference.
